# Supplementary material for: Application of Electronic Health Record Text Mining: Real-World Tolerability, Safety, and Efficacy of Adjuvant Melanoma Treatments
Source: Cancers (Basel). 2022 Nov 3;14(21):5426. doi: 10.3390/cancers14215426 (PMC9657798; doi:10.3390/cancers14215426)
Supplement: Supplementary file 1 [file cancers-14-05426-s001.zip › cancers-1929567-SM.pdf]

Supplementary Files

Supplementary File S1: Overview of queries for patient inclusion and data extraction in CTcue. (See separate excel file).

Supplementary File S2: Treatment limiting adverse events per individual patient for nivolumab (2a), pembrolizumab (2b), and dabrafenib plus trametinib (2c).

Supplementary file 2a

	**Nivolumab N = 9**									
Treatment-limiting adverse event per individual patient	**1**	**2**	3	4	5	6	7	8	9	Total
Colitis	1	1								2
Pneumonitis		1	1							2
Hepatitis				1	1					2
Thyroiditis				1	1					2
Myalgia				1						1
Myocarditis				1						1
Arthralgia						1				1
Hypocortisolism							1			1
Polymyalgia rheumatica								1		1
Meningitis									1	1
Adrenalitis									1	1
Total	1	2	1	4	2	1	1	1	2	

Supplementary file 2b

	**Pembrolizumab N = 3**			
Treatment-limiting adverse event per individual patient				Total
Colitis	1			1
Arthralgia		1		1
Myalgia		1		1
Pneumonitis			1	1
Total	1	2	1	

Supplementary file 2c

	**Dabrafenib plus trametinib N = 8**								
Treatment-limiting adverse event per individual patient	1	2	3	4	5	6	7	8	Total
Pyrexia	1	1	1	1	-	-	1	-	5
Chills	1	1	1	-	-	-	-	-	3
Nausea	1	1	1	-	-	-	-	-	3
Myalgia	1	-	-	-	-	1	-	-	2
Headache	1	-	-	-	-	-	-	-	1
Liver function disorders	1	-	-	-	-	-	-	-	1
Fatigue	-	1	-	-	-	-	-	1	2
Syncope	-	1	-	-	-	-	-	-	1
Skin disorder	-	-	-	1	1	1	1	-	4
Allergic reaction	-	-	-	-	1	-	-	-	1
Tachycardia	-	-	-	-	1	-	-	-	1
Decreased appetite	-	-	1	-	-	-	-	-	1
Arthralgia	-	-	-	-	-	1	-	-	1
Malaise	-	-	-	1	-	-	-	1	2
Total	6	5	4	3	3	3	2	2	
